# Skeletal muscle insulin resistance in salt-sensitive hypertension: role of angiotensin II activation of NF*κ*B

**DOI:** 10.1186/s12933-015-0211-6

**Published:** 2015-05-01

**Authors:** Ming-Sheng Zhou, Chang Liu, Runxia Tian, Akira Nishiyama, Leopoldo Raij

**Affiliations:** Department of Physiology, Liaoning Medical University, Jinzhou, China; Department of Endocrinology, Liaoning Medical University, Jinzhou, China; Hypertension/Nephrology Section, Miami VA Medical Center, Miami, FL USA; Department of Pharmacology, Kagawa University School of Medicine, Kagawa, Japan; Hypertension/Nephrology section, Vascular Biology Institute, University of Miami Miller School of Medicine, Miami, FL USA

**Keywords:** Angiotensin II, Hypertension, Insulin resistance, NFκB, Skeletal muscle

## Abstract

**Background:**

We have previously shown that in hypertensive Dahl salt-sensitive (DS) rats, impaired endothelium-dependent relaxation to acetylcholine and to insulin is mechanistically linked to up-regulation of angiotensin (Ang) II actions and the production of reactive oxygen species (ROS) and to activation of the proinflammatory transcription factor (NF)κB. Here we investigated whether Ang II activation of NFκB contributed to insulin resistance in the skeletal muscle of this animal model.

**Methods:**

DS rats were fed either a normal (NS, 0.5% NaCl) or high (HS, 4% NaCl) salt diet for 6 weeks. In addition, 3 separate groups of HS rats were given angiotensin receptor 1 blocker candesartan (ARB, 10 mg/kg/day in drinking water), antioxidant tempol (1 mmol/L in drinking water) or NFκB inhibitor PDTC (150 mg/kg in drinking water).

**Results:**

DS rats manifested an increase in soleus muscle Ang II content, ROS production and phosopho-IκBα/IκBα ratio, ARB or tempol reduced ROS and phospho-IκBα/IκBα ratio. Hypertensive DS rats also manifested a reduction in glucose infusion rate, impaired insulin-induced Akt phosphorylation and Glut-4 translocation in the soleus muscle, which were prevented with treatment of either ARB, tempol, or PDTC. Data from the rat diabetes signaling pathway PCR array showed that 8 genes among 84 target genes were altered in the muscle of hypertensive rats with the increase in gene expression of ACE1 and 5 proinflammatory genes, and decrease of 2 glucose metabolic genes. Incubation of the muscle with NFκB SN50 (a specific peptide inhibitor of NFκB) ex vivo reversed changes in hypertension-induced gene expression.

**Conclusion:**

The current findings strongly suggest that the activation of NFκB inflammatory pathway by Ang II play a critical role in skeletal muscle insulin resistance in salt-sensitive hypertension.

## Introduction

Hypertension and type 2 diabetic mellitus (T2DM) are two powerful risk factors for the development of cardiovascular diseases (CVD) [1,2]. Salt sensitivity of blood pressure and insulin resistance have been identified as key elements underlying the relationship between hypertension and T2DM. Excess dietary salt and caloric intake, as commonly found in westernized diets, are linked not only to increased blood pressure, but also to defective insulin sensitivity and impaired glucose homeostasis [3-5]. Insulin resistance is highly prevalent in the patients with essential hypertension, particularly those with salt-sensitivity [6]. However, there are still critical gaps in our knowledge of the mechanisms that lead to the development of insulin resistance in salt-sensitive (SS) hypertension.

Abnormal activation of renin-angiotensin system (RAS), oxidative stress, excessive dietary salt and fat intakes are factors that contribute to the development of insulin resistance [7-10]. Insulin stimulates glucose transport and vasodilation through the activation of phosphatidylinositol 3-kinase (PI3K) and nitric oxide (NO) signaling pathway [11,12]. It has been shown that angiotensin (Ang) II inhibits insulin stimulation of PI3K, thereby preventing the activation of downstream signaling molecules, including NO production in endothelial cells, and Glut-4 translocation in skeletal muscle cells [13,14]. We have recently shown in hypertensive Dahl SS (DS) rat, a paradigm of human SS hypertension characterized by cardiovascular injury and insulin resistance, that the upregulation of local (vasculature) Ang II activates the redox-sensitive transcription factor nuclear factor kappa (NFκB), which contributes to endothelial dysfunction, vascular inflammation, and the impairment of insulin-mediated vasorelaxation [13,15,16].

There is considerable evidence showing that insulin resistance is associated with low-grade inflammation [10,17-19]. NF κB is a primary regulator for proinflammatory gene expression and inhibition of the NFκB inflammatory activation has been shown to improve insulin sensitivity and cardiovascular injury in metabolic and hypertensive diseases [17,20]. The skeletal muscle is the largest insulin sensitive tissue and handles over 75% of the insulin-mediated glucose disposal in the body [21]. Therefore, we tested the hypothesis that in SS hypertension, the activation of NFκB inflammatory pathway by upregulation of endogenous Ang II/reactive oxygen species (ROS) impairs insulin sensitivity and insulin signaling in the skeletal muscle.

## Methods

### Animals and experimental protocols

The animals were housed in facilities accredited by the American Association for Accreditation of Laboratory Animal Care. The Institutional Animal Care and Use Committee at the Miami Veterans Affairs Medical Center approved the studies. Six-week-old DS male rats were purchased from Harlan Sprague-Dawley (Indianapolis, IN) and maintained under controlled conditions of light, temperature, and humidity. After 2 weeks of acclimatizing to the new environment, the rats were divided into 5 groups and treated for 6 weeks as follow: NS, fed 0.5% NaCl (normal salt) diet (n = 6); HS, fed 4% NaCl (high salt) diet (n = 7); HS/ARB: fed HS plus angiotensin receptor 1 blocker candesartan (ARB, 10 mg/kg/day in drinking water, n = 7); HS/Temp: fed HS diet plus antioxidant tempol (1 mmol/L in drinking water, n = 6); HS/PDTC, fed HS diet plus pyrrolidine dithiocarbamate (PDTC, 150 mg/kg in drinking water, n = 6), an inhibitor of NFκB activation. At the end of the study, the rats were euthanized by decapitation and the soleus muscles were harvested for determination of Ang II content, superoxide (O_2_^-^) production and PCR array gene expression.

### Determination of Ang II level

Ang II content in the soleus muscle was measured using the method established by Nishiyama et al and quantified with a competitive single antibody radioimmunoassay [22], using rabbit anti-Ang II antibody (Peninsula, Belmont, CA) and monoiodinated ^125^I-labeled Ang II (Amersham, Arlington Heights, IL). All samples were assayed in duplicates, and mean values were plotted against a curve generated by the Ang II standard.

### Determination of O_2_^–^ generation

The soleus muscle was isolated and cut into small pieces. Generation of O_2_^–^ in fresh tissue was determined by the chemiluminescence of lucigenin (5 μM), as previously described [23], and the results were expressed as counts/min/mg dry tissue. The chemiluminescence of lucigenin has been validated as a method in our previous studies to measure O_2_^-^ [23].

### Hyperinsulinemic-euglycemic clamp study and determination of intra-arterial pressure

A separate group of rats were used to determine mean arterial blood pressure (MAP) and glucose infusion rate, an index for metabolic insulin sensitivity, measured by the hyperinsulinemic-euglycemic clamp. The animals were fasted overnight before the experiments were conducted. The rats were anesthetized with pentobarbital sodium (50 mg/kg I.P.) and maintained at 37°C with a heating pad. The right femoral vein and left femoral artery were catheterized and used for monitoring blood pressure. The arterial catheter was connected to a pressure transducer. After a 60-min equilibration period, MAP was recorded by Powerlab (ADInstruments Inc, Colorado Springs CO). The rats were then used for a hyperinsulinemic-euglycemic clamp study. The right femoral vein and left femoral artery were used for glucose and insulin infusion and blood sampling, respectively. Baseline plasma samples were obtained, after which insulin (Sigma) at a constant rate of 30 mU · kg^–1^ · min^–1^ and glucose (17.5 g/100 ml saline) at varying infusion rates were continuously infused for 120 min. The blood glucose concentration, measured with an automatic blood glucose meter (Accu-Chek Advantage Blood Glucose Meter), was clamped at 5.5 mmol/L. Fasting plasma levels of insulin at baseline was measured by ELISA following the manufacture’s instructions (R&D Systems, Minneapolis, MN). Euglycemia was achieved by 60 min and maintained for 60 min. The glucose infusion rate was adjusted according to the blood glucose levels at 5-min intervals during the first 60-min period and, once stable, at 15-min intervals during the second 60-min period. The samples obtained over the second 60-min period were averaged and reported as the mean steady-state glucose infusion rate (mg · kg^–1^ · min^–1^) required for maintaining euglycemic conditions in the setting of hyperinsulinemia. After the completion of the euglycermic clamp, the soleus muscles were harvested and used for determination of the insulin signaling molecules.

### Western blot

The protein expression of IκBα, phospho (Ser 32)-IκBα, phospho (Ser 612)-IRS1, or phsopho (Ser 473)-Akt in the soleus muscle was determined by Western blot analysis. Briefly, after homogenization, protein concentration was determined by Bio-Rad protein assay. Thirty μg of protein were separated by SDS-PAGE and transferred to a nitrocellulose membrane. Transferred proteins were incubated overnight with specific polyclonal antibodies against IκBα, phospho (Ser 32)-IκBα, phospho (Ser 612)-IRS1 or phospho (Ser 473)-Akt (Cell Signaling). After washing, the blots were incubated with a secondary antibody and the signal was detected by luminol chemiluminescence followed by exposure to an autoradiography film. The membrane was reblotted for β-actin (Santa Cruz Biotechnology Inc.), to serve as a loading control. The data was normalized to β-actin and expressed as fold increase versus NS group.

### Isolation of membrane fraction and determination of Glut-4 expression

The soleus muscle was homogenized and centrifuged for 10 min. at 1,000 g at 4°C. The supernatant was saved; the pellet was resuspended in 1/3 of the initial volume, and centrifuged again at 1,000 g for 10 min. The two supernatant solutions were mixed and submitted to centrifugation at 41,000 g for one hour. The final pellet was resuspended in 100 μl of buffer as a total membrane fraction. Glut-4 expression was determined by Western blot and normalized by β-actin.

### Real-time PCR

Total RNA (2 μg) was extracted from the soleus muscle and reverse-transcribed using the SuperScript II RT First Strand Synthesis kit (Gibco, BRL), according to the manufacturer’s directions. Real-time PCR (RTq-PCR) for p22phox (Assay ID: Rn00577357_m1, Life Technologies), gp91phox (Assay ID: Rn00576710_m1) of nicotinamide adenine dinucleotide phosphate (reduced form, NADPH) oxidase subunits, suppressors of cytokine signaling 3 (SOCS3, Assay ID: Rn00585674-S1) and interleukin 6 (IL6, Assay ID:Rn00561420-m1) was performed in 20 μl reaction mixture containing an appropriately diluted (80 ng) cDNA solution, 0.1 μmol/L each primer, 0.2 μmol/L probe and PCR Master Mix assay kit (ABI) as previously described [7]. All PCR primers with fluorescence probe were ordered through life technologies (ABI) online system with assay ID. The relative expression of each mRNA was normalized by a housekeeping gene (GADPH), and expressed as fold increase vs. the NS group.

### PCR array for determination of mRNA profile of rat diabetes signaling pathway in the soleus muscle

The soleus muscles from DS rats fed a NS or HS diet for 6 weeks were dissected and cut into small pieces. The muscles from HS rats were incubated with SN50 (55 μg/ml, a cell-permeable peptide specific inhibitor of NFκB nuclear translocation) or SM50 (55 μg/ml, a mutated inactive control peptide, BIOMOL Research Lab. Inc. (Plymouth Meeting, PA)) in DMEM containing 0.1% BSA bubbled with 95% oxygen supplement for 120 min. The muscle was homogenated in 1 ml Trizol reagent (Life Technologies). One μg of RNA was converted to cDNA with random primers in a 20-μl reaction volume using a high capacity cDNA archive kit (C-3, Superarray). The cDNA was diluted to a volume of 100 μl, one μl cDNA was used for each primer set in the PCR Array according to the manufacturer’s protocol. Rat diabetes signaling pathway (PARN-023, Superarray) PCR array was used to determine a panel of diabetes gene expression. This PCR array includes 84 target genes related to obesity, insulin resistance, early onset of diabetes, complications from diabetes mellitus, and 12 control genes. Additionally, control genes are included in each array to control for genomic DNA contamination, RNA quality, housekeeping and general PCR performance. Data analysis was performed using the manufacturer’s integrated web-based software package for the PCR Array System using delta-delta Ct based fold-change calculations and normalized by a housekeeping control gene. Based on PCR array results, we further confirmed the expression of SOCS3 and IL 6 by RTq-PCR.

### Data analysis

The results were expressed as mean ± standard error of the mean (SEM). Statistical analyses were performed by ANOVA with Bonferonni’s correction for multiple comparisons. Significance was assumed at p < 0.05.

## Results

### MAP and metabolic parameters

**Figure 1:**
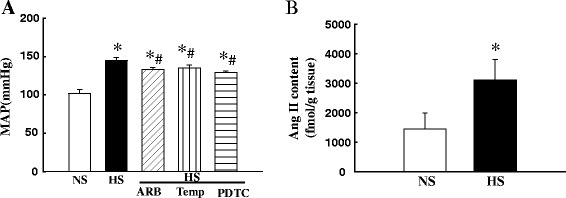
Mean arterial blood pressure (MAP, **A**) and Ang II content **(B)** in the soleus muscle of Dahl salt-sensitive (DS) rats. The data was expressed as mean ± SEM. *P < 0.05 vs. NS. #p < 0.05 vs. HS; N = 6-7.

High salt intake for 6 weeks significantly increased MAP (147 ± 6 vs. 103 ± 4 mmHg in NS; P < 0.05), as assessed by direct intra-arterial measurements. ARB candesartan (HS/ARB), antioxidant tempol (HS/Temp) or PDTC (HS/PDTC) treatment slightly but significantly reduced MAP (20-25% reduction, all p < 0.05). The rats however remained severely hypertensive (Figure 1A). Hypertensive DS rats manifested a tendency towards decreased body weight gain as compared with NS rats, but this did not reach statistical significance (384 ± 13 vs. 405 ± 9 g in NS, p < 0.1). The ARB, tempol or PDTC treatment did not affect body weight (data not shown). There were no significant differences in fasting plasma levels of insulin (3.6 ± 0.9 in HS vs. 3.3 ± 0.7 ng/ml in NS, p > 0.05) or glucose (95 ± 6 in HS vs. 91 ± 5 mg/dl in NS, p > 0.05) between HS and NS DS rats. Treatment with ARB, tempol or PDTC did not affect fast plasma levels of insulin or glucose (data not shown).

### Increased endogenous Ang II level in the soleus muscle of hypertensive DS rats

As shown in Figure 1B, Ang II concentration in the soleus muscle from hypertensive DS rats was significantly higher than in those in the normotensive DS rats (3145 ± 575 vs. 1405 ± 480 fmol/g tissue in NS, p < 0.05). To our knowledge, this is the first evidence showing an increase in endogenous Ang II level in the skeletal muscle of genetic SS hypertensive animal model.

### Increased O_2_^-^ production was linked to upregulation of Ang II action in the soleus muscle of hypertensive DS rats

**Figure 2:**
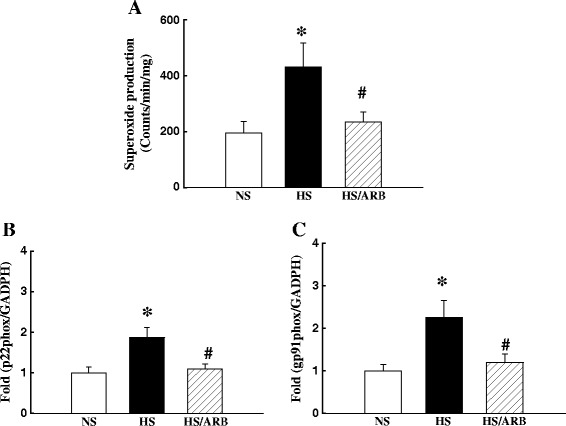
Superoxide (O_2_^-^) production **(A)** and the mRNA expression of nicotinamide adenine dinucleotide phosphate (reduced form, NADPH) oxidase subunits p22phox **(B)** and gp91phox **(C)** in the soleus of DS rats. O_2_^-^ production and the expression of p22phox and gp91phox were significantly increased in the muscle of hypertensive DS rats, compared with normotensive DS rats. ARB candesartan significantly reduced O_2_^-^ production and the mRNA expression of p22phox and gp91phox . *P < 0.05 vs. NS, #p < 0.05 vs. HS; N = 5-7.

We have previously shown that upregulation of Ang II was linked to increased vascular O_2_^-^ production in this animal model [16,23]. Hypertensive DS rats also had a significant increase in O_2_^-^ production in the soleus muscle, determined by lucigenin chemiluminescence, and ARB candesartan normalized O_2_^-^ production (Figure 2A). In addition, using RTq-PCR, we determined the expression of NADPH oxidase subunits, including p22phox and gp91phox in the soleus muscle. The mRNA expression of p22phox and gp91phox in HS rats was significantly increased by 87% and 125% respectively, compared with NS rats (all p < 0.05) and ARB treatment in HS-DS rats prevented the increase in the mRNA expression of p22phox and gp91phox (all p < 0.05, Figure 2B-2C).

### The treatment of ARB or tempol inhibited NFκB activation in the soleus muscle of hypertensive DS rats

**Figure 3:**
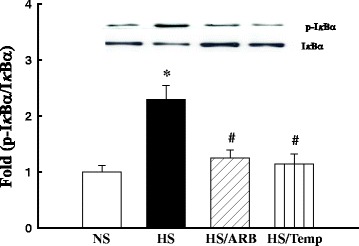
Protein expression of inhibitory kappa Bα(IκBα) and phospho (Ser32)-Iκbα in the soleus muscle of DS rats. The expression of IκBα was decreased, the ratio of IκBα/phospho-IκBα (Ser32) was increased in the muscle of hypertensive DS rats, which were significantly inhibited by the treatment of either angiotensin receptor 1 blocker (ARB) candesartan or antioxidant tempol. *P < 0.05 vs. NS, #p < 0.05 vs. HS. N = 4-5.

NFκB activation is initiated from the phosphorylation of IκB. Decreased IκB or increased ratio of phospho-IκB/IκB is an index of NFκB activation [24]. As shown in Figure 3, hypertensive DS rats exhibited a significant decrease in IκBα expression and increase in the ratio of phospho-IκBα/IkBα compared with normotensive DS rats (all p < 0.05). Either ARB candesartan or antioxidant tempol normalized IκBα or the ratio of phsopho-IkBα/IkBα, suggesting upregulation of endogeous Ang II/ROS participate in NFκB activation.

### The ARB candesartan, antioxidant tempol or inhibition of NFκB PDTC improved insulin sensitivity in hypertensive DS rats

**Figure 4:**
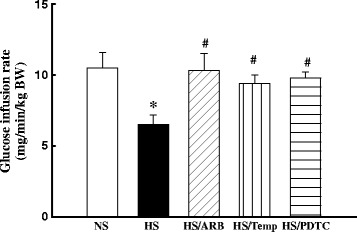
Effects of ARB, tempol or pyrrolidine dithiocarbamate (PDTC) on glucose infusion rate (determined by hyperinsulinemic euglycemic clamp) in DS rat. Glucose infusion rate to maintain plasma glucose at 5.5 mmol/l during insulin-infusion period was significantly reduced in hypertensive DS rat, and significantly improved by the treatment of either ARB, tempol or PDTC treatment. *p < 0.05 vs. NS, #p < 0.05 vs. HS; N = 6-7.

To determine the glucose infusion rate, an index of insulin sensitivity, we used the hyperinsulinemic-euglycemic clamp technique. As shown in Figure 4, the glucose infusion rate necessary to maintain plasma glucose levels at 5.5 mmol/L was significantly lower in HS rats when compared with NS rats (p < 0.05), indicating the presence of metabolic insulin resistance in hypertensive DS rats. The treatment with either ARB, tempol or PDTC significantly improved the glucose infusion rate (all p < 0.05).

### Protein expression of insulin signaling molecules

**Figure 5:**
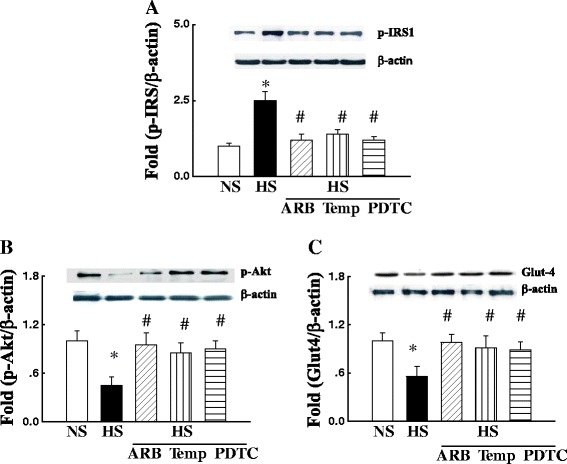
The protein expression of phospho (Ser 612)-insulin receptor substance 1 (IRS1, **A**), phospho (Ser 473) Akt **(B)** and Glut-4 in membrane fraction **(C)** in the soleus muscle of DS rats. The protein expression of phospho (Ser 612)-IRS1 was increased, and phospho (Ser 473) Akt and Glut-4 in the membrane fraction was significantly decreased in hypertensive DS rats, which was restored by the treatment of either ARB, tempol or PDTC treatment. *P < 0.05 vs. NS, #p < 0.05 vs. HS; N = 4-5.

It is well known that insulin induces glucose transport via activation of the IRS-1/PI3K/Akt/Glut-4 pathway [21]. The expression of phospho (Ser 612)-IRS-1 was significantly increased in HS group compared with NS group (p < 0.05) and was normalized by the treatment of either ARB, tempol or PDTC treatment (Figure 5A). Downstream molecules of the insulin activation of the PI3K pathway in the skeletal muscle include the phosphorylation of Akt and Glut-4 translocation in the membrane. As shown in Figures 5B&C, the protein expression of phospho-Akt at Ser473, and Glut-4 in membrane fraction was significantly reduced in the soleus muscle of hypertensive DS rats (all p < 0.05) and restored in the rats treated with either ARB, tempol or PDTC (Figure 5B&C).

### Effect of NFκB inhibition on gene profile related to onset, development and progression of diabetes in the soleus muscle of hypertensive DS rats

**Figure 6:**
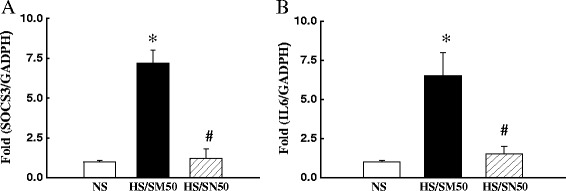
The mRNA expression of suppressors of cytokine signaling 3 (SOCS3, **A**) and interleukin 6 (IL6, **B**) was significantly decreased in the soleus muscle of hypertensive DS rats treated with control peptide SM-50. Inhibition of nuclear factor kappa B (NFκB) by specific inhibitor of SN-50 significantly reduced the mRNA expression of SOCS3 or monocyte chemoattractant protein-1 (MCP1). *P < 0.05 vs. NS, #p < 0.05 vs. HS/SM50; N = 4-5.

NFκB is a primary regulator of inflammatory responses by increasing transcriptional activity of at least 125 genes, most of which are proinflammatory [25]. Here we used the rat diabetes signaling pathway PCR array to determine key gene expression changes (84 target genes) in the soleus muscle. The PCR array includes genes that contribute to obesity, insulin resistance, the early onset of diabetes, and complications from diabetes. As shown in the Table 1, among the 84 target genes, 8 genes were altered in the soleus muscle of HS-DS rats treated with control peptide SM-50, compared with NS rats. Of the 8 genes whose expression was significantly altered, 6 increased including: angiotensin converting enzyme (ACE)1, 5 of proinflammatory genes including tumor necrosis factor (TNF)α, SOCS3, IL-6, monocyte chemoattractant protein-1 (MCP-1), and intracellular adhesion molecule-1 (ICAM-1). Additionally, 2 genes regulating glucose metabolic enzyme glycerol-3-phosphate dehydrogenase 1 and peroxisome proliferator-activated receptor coactivator (PCG)1 (the gene that regulate mitochondria biogenesis) were decreased. Pre-incubation with NFκB peptide inhibitor SN50 (HS-SN50) reversed gene expression changes in seven of the above mentioned genes, ACE1 was not significantly affected by SN50 treatment. In addition, we further confirmed that individual gene expression of SOCS3 and IL6 was significantly increased in hypertensive rats (HS-SM50) and reduced in hypertensive rats treated with NFκB inhibitor SN-50 by real-time PCR (Figure 6). These data suggest that activation of NFκB may play a role in the induction of skeletal muscle inflammation and insulin resistance in hypertension.

**Table 1:** **Profile gene by PCR array in skeletal muscle of DS rats (fold increase)**

**Gene**	**NS**	**HS/SM50**	**HS/SN50**
**ACE1**	1 ± 0.1	1.7 ± 0.2*	1.6 ± 0.2*
**MCP1**	1 ± 0.2	2.5 ± 0.6*	1.5 ± 0.4#
**ICAM1**	1 ± 0.1	8.2 ± 4.6*	2.9 ± 1.2*#
**IL6**	1 ± 0.2	10.1 ± 1.3*	1.7 ± 1.1*#
**TNFα**	1 ± 0.1	1.7 ± 0.4*	0.7 ± 0.2#
**SOCS3**	1 ± 0.1	7.3 ± 3.6*	1.5 ± 0.7#
**GPD1**	1 ± 0.1	0.6 ± 0.1*	1.1 ± 0.2#
**PGC1**	1 ± 0.2	0.4 ± 0.1*	1.2 ± 0.2#

Note: PCR: polymerase chain reaction; DS: Dahl salt-sensitive; ACE1: angiotensin converting enzyme 1; MCP1: monocyte chemoattract protein 1; ICAM1: intracellular adhesion molecule 1; IL6: interleukin 6, TNFα; Tumor necrosis factor a; SOCS3: suppressor of cytokine signaling 3; GPD1: Glycerol-3- phosphate dehydrogenase 1, PCG1: PPARγ coactivated factor 1. *p < 0.05 vs. NS; #p < 0.05 vs. HS-SM50. N = 4.

## Discussion

The present study demonstrates that the synthesis of Ang II is increased in the skeletal muscle of genetic SS hypertensive DS rats. Increased Ang II stimulation of NADPH oxidase-derived ROS activates NFκB inflammatory pathway, which in turn impairs insulin signaling and Glut-4 translocation in the skeletal muscle and induces systemic insulin resistance.

### Association of hypertension and insulin resistance: role of RAS

Diminished tissue sensitivity to insulin is a central feature of various pathological conditions termed the metabolic syndrome (MS) [1]. Insulin resistance and hypertension are key components of MS and often co-exist. Since patients with MS are commonly afflicted with cardiovascular morbidities, MS and CVDs share common pathways, such as, activated RAS, increased oxidative stress, defective glucose and lipid metabolism, low grade inflammation and endothelial damage [1,2].

The effects of the systemic RAS on blood pressure and glucose metabolism have been well demonstrated [9], and local tissue RAS in the skeletal muscle, vasculature, adipocytes and pancreas may also play an important role in the development of insulin resistance and vascular injury in diabetic and hypertensive diseases [14,26]. Ang II (via AT1R), the predominant component of RAS, induces insulin resistance through variety of mechanisms including inhibition of insulin signaling and insulin mediated glucose uptake in the skeletal muscle, decreased insulin secretion from pancreatic beta cells and alternation in adipocyte homeostasis [27,28]. In contrast to the Ang II/AT1R axis, other components of RAS, such as Ang (1-7), AT2R or AT4R may have alternate effects on insulin sensitivity and vascular function [29-31]. For example, Ang- (1-7) improves insulin sensitivity and pancreatic β cell survival in STZ-induced diabetic mice [31] and deletion of AT2R may reverse diabetes-induced endothelial function and vascular injury [30]. We have previously shown that in SS hypertension upregulation of local Ang II action (vasculature) induced endothelial dysfunction and vascular injury [13], here we further demonstrated that the blockade of Ang II by ARB candesartan improved skeletal muscle and systemic insulin sensitivity in hypertensive DS rats. Our data support the idea that activation of RAS is a common link between insulin resistance and vascular injury in SS hypertension.

We have to emphasize although it has been shown that ARBs are superior to other active antihypertensive agents for reduced incidence of new onset of TD2M in the patients with hypertension, the association of ARBs with diabetes risk may differ [32,33]. Some ARBs, for example, olmesartan may be associated with a slightly increased risk of diabetes mellitus [32]. The different actions of anti-diabetic effects by ARBs may be related to improvement of insulin sensitivity and other properties of ARBs such as activation of peroxisome proliferator-activated receptor gamma or insulin sensitizing effects [33,34].

### RAS/ROS in skeletal muscle insulin resistance

It has been shown that skeletal muscle cells contain many components of RAS, including angiotensinogen, Ang I and Ang II, ACE1, AT1R and AT2R [21,35]. The present study showed that Ang II content was increased in the skeletal muscle of hypertensive DS rats. As the expression of ACE1 was also increased in the skeletal muscle of hypertensive DS rats (Table 1), the increased Ang II content may mainly be due to an increased rate of in situ synthesis of Ang II. Therefore, Ang II has prooxidant effects, as the upregulation of Ang II increased ROS production through stimulation of NADPH oxidase in the muscle (Figure 2) [36].

An increasing body of evidence supports the role of Ang II in the multifactorial etiology of skeletal muscle insulin resistance [36-38]. Acute infusion of Ang II into the interstitial space of skeletal muscle has been shown to impair insulin-mediated glucose uptake, which was independent of alteration in blood flow [39]. Chronic infusion of Ang II in the rat was associated with diminution of whole body glucose disposal and reduced skeletal muscle glucose uptake, likely due to increased ROS production [3,40]. Furthermore, in cultured L6 myotubes, Ang II stimulated serine-phosphorylation of IRS-1 [36]. Phosphorylation of IRS-1 at specific serine residues inhibited insulin stimulation of tyrosine phosphorylation, subsequently inhibiting downstream PI3K signaling [14].

The present study demonstrated that upregulation of endogenous Ang II-induced ROS impaired insulin signaling and Glut-4 translocation in the skeletal muscle of hypertensive rats and resulted in systemic insulin resistance. Since Ang II/ROS (via NADPH oxidase) is present in both skeletal myocytes and the vascular tissue of arterioles that supply blood to these myocytes [26,35], a large number of studies have shown a relationship between microvascular dysfunction and skeletal muscle insulin resistance [41-43]. The present study has some limitations that we did not investigate the effects of insulin on microvascular function in the skeletal muscle. However, based on our previous findings that ARB or antioxidant tempol improved endothelium function and insulin-induced vasorelaxation in the aorta of this animal model [13,15], it is reasonable to speculate that improvement of skeletal muscle insulin resistance by the ARB or antioxidant tempol may be, at least in part, attributed to their hemodynamic action in skeletal muscle [26,36,40].

### Ang II and ROS converge on NFκB to inhibit insulin signaling

It is increasingly recognized that chronic inflammation plays a critical role in the pathogenesis of hypertension, insulin resistance and atherosclerotic diseases [44,45]. NFκB is a redox-sensitive transcript factor that regulates the transcription of a large number of proinflammatory genes. Ang II, oxidative stress and proinflammatory cytokines are the factors that induce NFκB activation [25]. Ang II binding to AT1R activates NFκB in a ROS-dependent manner. ROS activates IκB kinase (IKK) to induce IκB phosphorylation, resulting in the activation of NFκB [25,46]. Here we showed that hypertensive DS rats exhibited a decreased IκB and increased the ratio of phospho-IκBα/IκBα, which was prevented by either ARB candesartan or antioxidant tempol, suggesting involvement of Ang II/ROS in activation of NFκB in skeletal muscle of this animal model. Furthermore, inhibition of NFκB using SN50 reversed gene profile related to development of insulin resistance and diabetes and improved skeletal muscle insulin signaling. Moreover, a number of the genes such as proinflammatory genes of TNFα, IL6 and COCS3, which were increased in the muscle of hypertensive DS rats (Table 1), have been shown to play a fundamental role in the pathogenesis of insulin resistance and T2DM [47,48]. Therefore, our data support the idea that in SS hypertension, Ang II and ROS converge on NFκB signaling, which in turn induces skeletal muscle inflammation and insulin resistance.

In summary, clinical and experimental studies have shown that the inhibition of Ang II by ACE inhibitors or ARBs or antioxidants improve insulin sensitivity and glycemic control in diabetic patients and animals and reduce the incidence of new onset type 2 diabetes [33,39]. The present study demonstrated that increased endogenous Ang II/ROS activation of NFκB inflammatory pathway impairs insulin signaling and Glut-4 transolcation in the skeletal muscle of SS hypertension. The current finding, in conjunction with our recent studies [13,15] in the effects of ARB, antioxidant or NFκB inhibition on endothelial function, vascular inflammation and CV injury strongly suggest that Ang II/ROS activation of NFκB is an important link among hypertension, vascular injury and insulin resistance.

## Abbreviation

Ang II: Angiotensin II
ACE: Angiotensin converting enzyme
ARB: Angiotensin receptor 1 blocker
CVD: Cardiovascular disease
DS: Dahl salt-sensitive
IRS1: Insulin receptor substance 1
IL6: Interleukin 6
ICAM1: Intracellular adhesion molecule-1
MAP: Mean arterial blood pressure
MCP-1: Monocyte chemoattractant protein-1
NADPH: Nicotinamide adenine dinucleotide phosphate (reduced form)
NO: Nitric oxide
NFκB: Nuclear factor kappa B
PI3K: Phosphatidylinositol 3-kinase
PDTC: Pyrrolidine dithiocarbamate
ROS: Reactive oxygen species
RAS: Renin-angiotensin system
SS: Salt-sensitive
O_2_^-^: Superoxide anion
SOCS3: Suppressors of cytokine signaling 3
TNFα: Tumor necrosis factor α
T2DM: Type 2 diabetic mellitus
